# Blood–Ocular Barrier Dysfunction in Uveitis: A Bidirectional Model Linking Pathogenesis, Clinical Monitoring, and Therapeutic Opportunities

**DOI:** 10.3390/medsci14020290

**Published:** 2026-06-05

**Authors:** Yijin Chen, Mingming Yang, Yaru Zou, Jing Zhang, Kyoko Ohno-Matsui, Koju Kamoi

**Affiliations:** Department of Ophthalmology and Visual Science, Graduate School of Medical and Dental Sciences, Institute of Science Tokyo, Tokyo 113-8519, Japan; cccccyi960610@163.com (Y.C.); yangmm-12@outlook.com (M.Y.); alicezouyaru519@gmail.com (Y.Z.); zhangj.c@foxmail.com (J.Z.); k.ohno.oph@tmd.ac.jp (K.O.-M.)

**Keywords:** uveitis, blood–ocular barrier, blood–retinal barrier, inflammation, vascular permeability, clinical monitoring, fluorescein angiography, macular edema, aqueous flare, relapse

## Abstract

Uveitis is a heterogeneous group of intraocular inflammatory diseases and an important cause of visual impairment worldwide. Although current treatments mainly target inflammation, many patients develop chronic or recurrent disease, suggesting that inflammation control alone may not fully restore intraocular homeostasis. Increasing evidence highlights the blood–ocular barrier (BOB), including the blood–retinal barrier and blood–aqueous barrier, as a key regulator of the intraocular microenvironment. This review aims to summarize the bidirectional interaction between intraocular inflammation and blood–ocular barrier dysfunction in uveitis, and to highlight the clinical significance of barrier dysfunction in disease monitoring and management. In addition, this review discusses the potential value of incorporating barrier assessment into dynamic disease evaluation and relapse-aware management strategies. Recent studies suggest that inflammation and BOB dysfunction are bidirectionally linked. Inflammatory mediators disrupt barrier integrity, while barrier breakdown facilitates immune cell infiltration and further amplifies inflammation, forming a self-reinforcing cycle that may drive disease persistence. Importantly, BOB dysfunction also has clinical implications. Findings such as aqueous flare, macular edema on optical coherence tomography, and vascular leakage on fluorescein angiography reflect barrier status and can serve as dynamic indicators for disease monitoring. Persistent abnormalities despite reduced inflammatory cell activity may indicate incomplete barrier recovery or subclinical inflammation, helping to explain discordant clinical findings and the relapse-prone nature of uveitis. Rather than viewing BOB dysfunction solely as a pathological consequence of inflammation, this review highlights the potential clinical value in disease assessment and management. Barrier-related findings may provide additional information beyond conventional inflammatory evaluation, particularly in cases where inflammatory cell activity appears controlled, but underlying barrier alteration persists. Incorporating barrier assessment into monitoring may help interpret discordant clinical findings, improve evaluation of disease control, and support a more relapse-aware management strategy in uveitis. In addition, therapeutic approaches aimed at restoring barrier integrity may provide a more comprehensive strategy for achieving sustained remission and reducing recurrence risk.

## 1. Introduction

Uveitis is an intraocular inflammatory disease that can occur at any age and mainly affects the uveal tract. Clinically, it represents a heterogeneous group of disorders with diverse etiologies and disease courses, and can be broadly classified into infectious and non-infectious types [1,2]. Its main symptoms include eye redness, pain, photophobia, floaters, and blurred vision. Depending on the severity and anatomical involvement, visual impairment may range from transient blurring to irreversible vision loss. If not treated in time, uveitis may lead to cataract, glaucoma, macular edema, retinal detachment, optic nerve damage, and even vision loss [2]. Studies suggest that it is an important cause of preventable blindness, particularly among people of working age [3].

Increasing evidence suggests that dysfunction of the blood–ocular barrier may play a central role in this process [4,5]. It is essential for maintaining a stable intraocular environment [6,7]. Previous studies have demonstrated that inflammation can disrupt barrier integrity, leading to increased permeability and allowing inflammatory cells and mediators to enter the eye, thereby contributing to the development of uveitis [5,8]. Consequently, corticosteroids remain the mainstay of treatment [9,10], with the aim of suppressing inflammation while minimizing steroid-related adverse effects. Although this approach is generally effective in controlling acute inflammatory activity, a substantial proportion of patients develop chronic disease or experience recurrent episodes after treatment. It has been reported that approximately 40–60% of patients develop a chronic or recurrent disease course, and a subset of these cases may become treatment-resistant [2,11,12]. Repeated inflammatory episodes can progressively damage ocular tissues, ultimately leading to irreversible vision loss. In addition, these patients often require long-term treatment and follow-up, imposing a considerable psychological and financial burden and significantly affecting quality of life [13].

These observations suggest that the pathogenesis of uveitis is more complex than traditionally assumed. Recent studies indicate that uveitis does not follow a unidirectional mechanism, but rather involves a more complex process characterized by a vicious cycle [5,8]. This review aims to summarize current evidence regarding the interaction between intraocular inflammation and blood–ocular barrier dysfunction in uveitis, with particular emphasis on its clinical implications in disease monitoring and management. In addition, this review discusses the potential value of barrier assessment in interpreting persistent or discordant clinical findings and supporting relapse-aware management strategies. The review first outlines the structure and physiological function of the blood–ocular barrier, followed by discussion of barrier alteration in ocular diseases and uveitis, and finally summarizes current clinical assessment methods, therapeutic implications, and future research directions.

## 2. Methodology

Relevant literature was primarily identified through searches of PubMed. Additional references were obtained from related articles and reference lists when relevant to the topic. Publications from approximately 2010 to 2025 were mainly included, while several earlier landmark studies, particularly those related to the structure and physiological function of the blood–ocular barrier, were also reviewed.

The literature search was performed using combinations of keywords including “uveitis”, “blood–ocular barrier”, “blood–retinal barrier”, “blood–aqueous barrier”, “barrier dysfunction”, “vascular permeability”, “inflammation”, “cytokines”, “macular edema”, “aqueous flare”, and “fluorescein angiography”. Article selection was based on relevance to the topic, with particular emphasis on studies addressing mechanisms of barrier disruption, bidirectional interactions between inflammation and barrier dysfunction, clinical assessment of barrier-related changes, and therapeutic implications in uveitis and related retinal diseases.

## 3. Structure and Function of Blood–Ocular Barrier in Health

**Figure 1:**
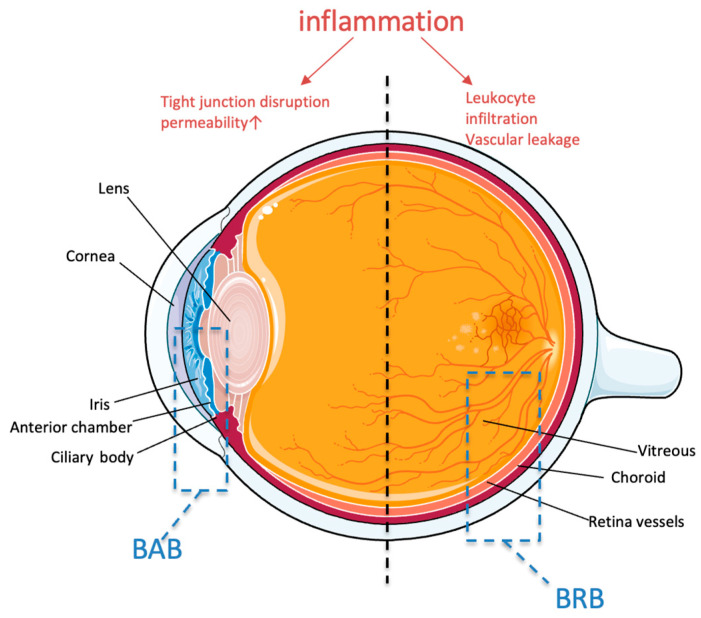
Structure and inflammation-associated disruption of the blood–ocular barrier.

The eye is an immune privilege organ, showing high immune tolerance and stringent regulation of inflammatory responses [14,15]. This immune privilege depends on a specialized stable microenvironment that is separated from the systemic circulation, and is maintained by blood–ocular barrier [16,17,18]. The blood–ocular barrier is a functional system formed by anatomical structures and tight junctions between ocular cells [19], it includes the blood–aqueous barrier (BAB), located in the anterior segment, and the blood–retinal barrier (BRB), located in the posterior segment [20]. BAB is mainly formed by tight junctions between nonpigmented epithelial cells of the ciliary epithelium and iris vascular endothelial cells [21]. Its main function is to keep the aqueous humor low in proteins and cells, which helps provide a clear and stable environment for the structures of the anterior segment [22]. BRB can be further divided into the inner and the outer BRB [23]. The inner BRB (iBRB) is formed by tight junctions between retinal capillary endothelial cells [19,24], and the BRB limits the paracellular movement of blood-derived macromolecules and immune cells, preventing inflammatory mediators from entering the neural retina in a non-specific manner [17,25]. The blood–ocular barrier normally limits blood antigens, immune cells, and inflammatory factors from entering the eye, which helps prevent unnecessary immune activation [26]. The BRB also plays an important role in maintaining the stable microenvironment required for normal retinal function by regulating the exchange of nutrients, ions, and metabolic waste between the circulation and the retina [4]. The anatomical structure of the blood–ocular barrier and the general processes involved in inflammation-associated barrier disruption are summarized in Figure 1.

Beyond acting as a physical barrier, the cells that form the blood–eye barrier also take part in immune regulation inside the eye [14,27]. The ciliary epithelial cells and iris vascular endothelial cells that form the BAB express several immune-regulating molecules, such as transforming growth factor-β (TGF-β), interleukin-10 (IL-10), programmed death ligand-1 (PD-L1), and Fas ligand (FasL) [28,29]. Through these molecules, they can suppress excessive immune activation and limit the spread of inflammatory mediators into the anterior chamber [28,30]. When the BAB remains intact, it helps keep the aqueous humor low in proteins and inflammatory cells [31]. During systemic inflammation, a functional BAB can help maintain a relatively stable and controlled microenvironment in the anterior segment [32]. Retinal pigment epithelial (RPE) cells which form outer BRB also actively participate in immune regulation inside the eye through several mechanisms [33]. First, under physiological conditions, RPE cells continuously secrete a variety of immunosuppressive factors such as TGF-β, IL-10, and α-melanocyte-stimulating hormone (α-MSH) [29]. These molecules can suppress T cell activation and promote the formation of regulatory T cells (Treg), thereby maintaining a local immune-tolerant environment [33]. Second, RPE cells express immune regulatory molecules on their surface, such as FasL and PD-L1 [29]. These molecules can induce apoptosis or functional suppression of activated T cells that enter the eye, limiting the further spread of inflammatory responses [34]. In addition, RPE cells can also regulate intraocular immunity through physiological pathways. By maintaining low expression of major histocompatibility complex class II molecules (MHC-II), RPE cells reduce antigen-presenting activity [35] and regulate the complement system to participate in immune regulation. Thus, the integrity of the blood–eye barrier is essential for maintaining intraocular homeostasis and normal retinal function.

## 4. Blood–Ocular Barrier Disruption in Ocular Diseases

The blood–ocular barrier can be disrupted by various reasons, such as inflammation, injury and induced ocular hypotony [36,37], leading to increased vessel permeability and loss of tight junctions, allowing proteins and immune cells to leak into the ocular areas and promoting disease development [38,39]. This has been well documented in many ocular diseases including diabetic retinopathy, uveitis, age-related macular degeneration and retinal vein occlusion [40,41].

### 4.1. Barrier Breakdown in Retinal Diseases

Blood–retinal barrier dysfunction is a common pathological feature in many retinal diseases and is often closely associated with inflammation, vascular instability, and retinal edema. Among these diseases, diabetic retinopathy has been one of the most extensively studied models of inflammation-associated barrier breakdown.

In diabetic retinopathy, breakdown of the blood–retinal barrier has been proposed as an early and important event in disease progression and is closely linked to chronic inflammation [40,42]. Long-term high blood glucose activates multiple inflammatory pathways, leading to dysfunction of retinal endothelial cells and promotes the adhesion and migration of inflammatory cells into retinal tissue [43,44]. Many inflammation-related cytokines and chemokines, including TNF-α, IL-1β, IL-6, and CCL2, were greatly increased in the aqueous and vitreous levels of patients with diabetic retinopathy [45]. The increase in these molecular levels is directly related to the disturbance of expression and localization of tight junction proteins [46]. As a result, the integrity of the blood–retinal barrier is disrupted, and vascular permeability is increased [43]. Increased barrier permeability promotes the entry of plasma proteins and immune cells into retinal tissue [47], thereby further amplifying local inflammation and establishing a persistent inflammatory microenvironment [42]. This process not only causes vascular leakage and macular edema but also promotes progressive damage to the retinal microvasculature [47]. Accordingly, in diabetic retinopathy, blood–retinal barrier impairment is both a consequence of inflammation and an important driver of ongoing disease progression.

Apart from diabetic retinopathy, BRB dysfunction is also observed in a wide range of ocular diseases, particularly in retinal disorders characterized by inflammation and pathological neovascularization [48]. In age-related macular degeneration and retinal vein occlusion, breakdown of the blood–retinal barrier is recognized as an important mechanism underlying vascular leakage, macular edema, and vision loss [19,49]. In these conditions, local hypoxia, oxidative stress, and inflammation act together on retinal vascular endothelial cells [50], leading to reductions in tight junction proteins and endothelial integrity disruption [4,51]. Many inflammatory factors and vascular-related molecules are confirmed to play important roles in this process [52]. Among them, vascular endothelial growth factor (VEGF) and pro-inflammatory cytokines are especially important drivers of barrier disruption [53,54].

### 4.2. Blood–Ocular Barrier Dysfunction in Uveitis

#### 4.2.1. Inflammation-Driven Blood–Ocular Barrier Disruption

In previous studies, inflammation was predominantly considered the initial cause of blood–ocular barrier disruption [5,55], and in classical theory of uveitis pathogenesis, inflammation occurs before blood–ocular barrier breakdown [56,57]. Studies show that inflammatory cytokines are key mediators of ocular inflammation and can directly disrupt the blood–retinal barrier, leading to vascular leakage and macular edema [5,47]. Among these cytokines, TNF-α and IL-1β are two of the most well-studied inflammatory mediators involved in endothelial barrier dysfunction [4]. Studies have shown that TNF-α can activate intracellular signaling pathways such as NF-κB in retinal endothelial cells, leading to reduced expression and disorganization of tight junction proteins including ZO-1, occluding, and claudin-5 [25]. These changes disrupt intercellular junctions between endothelial cells and increase vascular permeability. In addition, TNF-α has been reported to induce the expression of endothelial adhesion molecules such as ICAM-1 and VCAM-1 [58] thereby promoting leukocyte adhesion to vascular endothelium and facilitating inflammatory cell migration across the vascular barrier. Also, in in vitro blood–retinal barrier models, treatment with the pro-inflammatory cytokine TNF-α can directly impair endothelial barrier function [51], as evidenced by decreased trans endothelial electrical resistance (TEER) and increased permeability, together with disruption of tight junction structures [51,59]. Meanwhile, TNF-α treatment also significantly induces phosphorylation of Janus kinase 1 (JAK1), and inhibition of the JAK1 signaling pathway can partially restore TEER and reduce permeability [55], suggesting that JAK1 plays a key role in barrier disruption. Furthermore, modulation of intracellular 3′,5′-cyclic adenosine monophosphate (cAMP) levels shows that increasing cAMP attenuates TNF-α-induced barrier dysfunction, whereas decreasing cAMP aggravates the damage [51], indicating that this process is, to some extent, regulatable.

Similarly, IL-1β has also been shown to disrupt endothelial barrier function through several molecular mechanisms. Studies indicate that IL-1β can activate signaling pathways such as MAPK and NF-κB, which induce inflammatory gene expression and alter endothelial cell structure [60]. In addition, IL-1β has been reported to promote cytoskeletal rearrangement and actin stress fiber formation [61], which can destabilize tight junction complexes and further impair endothelial barrier integrity [62]. These structural changes contribute to increased vascular permeability and leakage of plasma components into retinal tissue [4]. Other inflammatory mediators such as IL-6, IL-8, IL-17, and CCL2 may also contribute to barrier dysfunction through overlapping pathways [4,63].

Besides the above cytokine-related signaling pathway, other mechanisms may also participate in inflammation-induced barrier disruption. Oxidative stress generated during inflammatory responses can also interrupt endothelial cells and tight junction structures, thereby aggravating barrier dysfunction [5,64]. In addition, increased expression of endothelial adhesion molecules can enhance leukocyte adhesion and trans endothelial migration, which further amplifies local inflammatory responses and contributes to progressive barrier breakdown [4].

Inflammation can also affect the stability of the blood–aqueous barrier. Studies have shown that various pro-inflammatory cytokines can alter the tight junctions of iris vascular endothelial cells and ciliary epithelial cells [65], thereby increasing the permeability of the blood–aqueous barrier [66]. In addition, inflammatory mediators can activate signaling pathways in endothelial cells and induce cytoskeletal rearrangement, which further weakens the integrity of cell–cell junctions and promotes the leakage of plasma components into the aqueous humor [66,67]. These findings suggest that in the inflammatory environment, like during uveitis, the blood–aqueous barrier, like the blood–retinal barrier, can be directly affected by inflammatory cytokines, leading to an overall decline in the stability of the blood–ocular barrier [68,69].

#### 4.2.2. Experimental Evidence Supporting Barrier Disruption in Uveitis

In vivo studies provide further evidence supporting these mechanisms. In experimental autoimmune uveitis (EAU) models, increased levels of inflammatory cytokines have been demonstrated by multiple experimental approaches [5]. Single-cell RNA sequencing (scRNA-seq) analysis shows that inflammation-related gene expression is significantly upregulated in multiple retinal cell types [70], such as Müller cells and retinal pigment epithelial cells, together with increased infiltration of immune cells including CD45^+^ leukocytes and Th17 cells [71,72], indicating activation of local inflammatory responses in the retina [70]. Moreover, studies shows that the levels of pro-inflammatory mediators, including TNF-α, IL-1β, IL-6, IL-8, IL-17, and the chemokine CCL2, are significantly elevated during the active uveitis [73], and are consistent with the extent of immune cell infiltration and tissue damage, suggesting that the expression of these pro-inflammatory factors is closely associated with disease activity.

#### 4.2.3. Barrier Dysfunction as an Early Event in Inflammation

However, the relationship between inflammation and the blood–ocular barrier may be more complex than described above. The blood–ocular barrier is a key structure for maintaining immune privilege and microenvironmental stability within the eye. Once its integrity and function are compromised, it not only weakens the restriction of immune cells and inflammatory mediators, but may also fundamentally alter the immune regulatory environment of the eye, thereby creating conditions for the development of inflammation. Based on this understanding, an increasing number of studies have begun to challenge the traditional one-way model in which inflammation precedes barrier breakdown, suggesting that blood–ocular barrier dysfunction may occur at an early stage of disease and contribute to the initiation and progression of inflammation.

Studies based on the experimental autoimmune uveitis (EAU) model have shown that, by combining vascular permeability assays with real-time observation of leukocyte behavior, it is possible to track the sequence of events between blood–retinal barrier (BRB) dysfunction and inflammatory cell infiltration [57]. Specifically, in the early stage of disease, leukocytes first adhere to the retinal vascular endothelium, and this is already accompanied by increased BRB permeability. In contrast, the actual transmigration of leukocytes across the endothelium into retinal tissue occurs at a later stage. This temporal pattern suggests that BRB dysfunction may not merely be a consequence, but an early event that facilitates subsequent inflammatory cell infiltration [57].

In addition, more recent studies have shown that disruption of barrier structure—such as the breakdown of tight junctions and cytoskeletal reorganization—can facilitate cell migration across the barrier, thereby contributing to the initiation and progression of inflammation [74]. Studies showed that under inflammatory conditions, leukocyte rolling, adhesion, and trans endothelial migration are significantly increased [75], accompanied by upregulation of endothelial adhesion molecules such as ICAM-1 and VCAM-1 [76], thereby enhancing leukocyte–endothelial interactions and promoting their entry into retinal tissue [77]. Furthermore, vascular permeability assays show that, with increased leukocyte infiltration, retinal vascular leakage is markedly aggravated, indicating that the extent of barrier damage is consistent with the degree of leukocyte infiltration.

Taken together, these processes are increasingly recognized as a key step preceding inflammatory cell entry into the retina, supporting the idea that barrier impairment is not merely a consequence of inflammation, but may also play an active role in driving its development [74,78].

In addition, single-cell transcriptomic analyses have shown that, before substantial infiltration of inflammatory cells into the retina, barrier-associated cells such as Müller glia and retinal pigment epithelial (RPE) cells already exhibit upregulation of inflammation-related genes and functional changes [70]. These findings suggest that these cells may be involved in early immune activation processes rather than acting solely as passive responders to inflammation.

In addition to uveitis models, studies in other retinal diseases further support this hypothesis. In diabetic retinopathy, blood–retinal barrier dysfunction is considered an early pathological event and has been closely associated with subsequent inflammatory responses and neurovascular unit impairment [79,80]. Also, some studies suggest that non-inflammatory factors, such as neural regulation and metabolic alterations [81,82], can influence barrier function even in the absence of overt inflammation, thereby affecting the regulation of immune cell entry into the retina. Although these findings are not all directly derived from uveitis, they support, at the mechanistic level, the idea that barrier dysfunction may occur before or independently of inflammation.

Importantly, this bidirectional interaction between inflammation and barrier dysfunction represents a key conceptual shift from the traditional linear model and provides a new framework for understanding the pathogenesis of uveitis. However, it should be noted that the current evidence supporting this conceptual framework remains incomplete. Much of the available data is derived from experimental models, while direct clinical evidence, particularly from prospective human studies, is still limited. These limitations should be taken into account when interpreting this bidirectional model.

#### 4.2.4. Bidirectional Interaction Between Inflammation and Barrier Dysfunction

**Figure 2:**
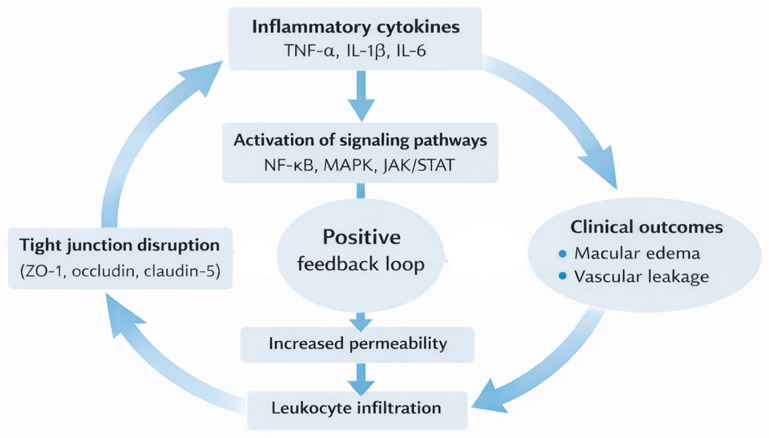
Bidirectional interaction of inflammation and blood–ocular barrier dysfunction in uveitis.

Taken together, current evidence suggests that the relationship between inflammation and blood–ocular barrier dysfunction is not a simple linear cause–effect process, but rather a mutually reinforcing positive feedback loop. Specifically, inflammation can disrupt barrier integrity while barrier dysfunction in turn facilitates immune cell recruitment and further amplifies inflammatory responses, forming a self-reinforcing cycle, as illustrated in Figure 2. As this process continues, uveitis may become more prone to chronicity and recurrence into a chronic or relapsing disease. This conceptual shift from a linear to a bidirectional model provides a useful framework for understanding uveitis pathogenesis and highlights the potential value of strategies aimed at protecting and restoring barrier function.

### 4.3. Current Evidence Gaps and Future Research Priorities

Despite increasing recognition of blood–ocular barrier (BOB) dysfunction in uveitis, important limitations remain in the current body of evidence [2]. Notably, most mechanistic and experimental insights are derived from studies focusing on the blood–retinal barrier (BRB), particularly those based on experimental autoimmune uveitis (EAU) models and in vitro systems [83,84]. While these studies have provided valuable information on cytokine-mediated tight junction disruption, leukocyte–endothelial interactions, and vascular permeability changes, their findings may not fully capture the complexity of human disease.

In contrast, evidence specifically addressing the blood–aqueous barrier (BAB) remains relatively limited. Although clinical observations such as aqueous flare measurements suggest increased BAB permeability during active inflammation, the underlying molecular mechanisms and their temporal relationship with disease progression are less well characterized [85,86]. Moreover, most available data are derived from cross-sectional or experimental studies, while prospective human studies evaluating barrier dynamics over the disease course are still lacking.

Another important limitation is the insufficient evidence supporting causal inference. While numerous studies have demonstrated associations between inflammatory mediators and barrier disruption, it remains unclear whether barrier dysfunction represents an initiating event or a secondary consequence in different subtypes of uveitis. Similarly, although barrier-related parameters such as fluorescein leakage and macular edema are widely used to reflect disease activity, their value in predicting relapse or guiding treatment decisions has not been systematically validated [87].

These gaps highlight the need for future research focusing on clarifying the role of BAB in uveitis pathogenesis, establishing longitudinal clinical cohorts to evaluate barrier function dynamics, strengthening causal inference through integrative experimental and clinical approaches, and exploring the potential of barrier-based biomarkers in relapse prediction and personalized management. Addressing these limitations will be essential to translate current mechanistic insights into clinically meaningful applications.

## 5. Clinical Assessment of Blood–Ocular Barrier Dysfunction

Barrier dysfunction does not stay only at the molecular or cellular level but also leads to observable clinical changes [31]. Previous studies show that barrier dysfunction is not a discrete event but a dynamic process throughout disease onset, progression, and relapse [4], so it causes not only structural damage but also measurable clinical changes at different disease stages [88,89]. These changes make barrier status an important clinical assessment in uveitis, and related tests can reflect disease activity and progression [90]. Currently, several clinical markers are used to indirectly reflect barrier function and are closely related to disease activity [91]. The major clinical modalities used to assess blood–ocular barrier dysfunction in uveitis are summarized in Table 1.

### 5.1. Aqueous Flare Measurement

Aqueous flare is the scattering of light within the anterior chamber, caused by an increased level of protein [31]. Clinically, it is assessed using a slit-lamp examination or a laser flare photometer. Normally, the blood–aqueous barrier functions to keep plasma proteins out of the aqueous humor [90]. As a result, protein levels there are very low, and little to no flare is detectable [92]. However, if the permeability of this barrier increases, plasma proteins can leak into the aqueous humor [31]. This leakage raises the protein concentration, which in turn strengthens the light scattering signal and leads to an observable increase in aqueous flare [31]. Therefore, the presence and intensity of an aqueous flare primarily indicate the functional status of the blood–aqueous barrier. It is important to note that it does not directly correspond to the number of inflammatory cells present.

In patients with uveitis, multiple clinical studies have confirmed a positive correlation between aqueous flare levels and the degree of inflammatory activity [97]. Importantly, flare can remain elevated in some cases of chronic or recurrent disease, even when the number of inflammatory cells has significantly decreased [98]. This finding suggests that recovery of the blood–aqueous barrier may lag behind the apparent resolution of cellular inflammation.

From a clinical perspective, persistent elevation of aqueous flare despite reduced inflammatory cell counts may indicate incomplete restoration of barrier integrity or the presence of residual subclinical inflammation.

### 5.2. Imaging of Macular Edema

Macular edema refers to the thickening of the retina in the macula caused by abnormal accumulation of fluid [99]. Clinically, it is mainly observed and measured using optical coherence tomography (OCT) [100]. Macular edema is one of the most common and clinically significant complications in uveitis [99]. Under normal conditions, the blood–retinal barrier regulates the permeability of retinal blood vessels. It maintains fluid balance in the retina [101]. This ensures the structural and functional integrity of the macula [19]. When the blood–retinal barrier is damaged, retinal blood vessels become more permeable [101]. Plasma components and fluid leak into the retinal tissue. This eventually leads to cystoid or diffuse macular edema [102]. Macular edema is considered a direct structural reflection of blood–retinal barrier breakdown [102]. In uveitis, macular edema is more common in patients with moderate to severe disease or a longer disease course [93]. It is closely associated with decreased visual function [101,103]. Moreover, persistent macular edema often indicates long-term damage to the blood–retinal barrier. Even when inflammatory activity appears to decrease, these structural changes may persist, reflecting incomplete restoration of barrier integrity [104]. Accordingly, persistent macular edema may also reflect ongoing blood–retinal barrier dysfunction despite apparent improvement of inflammation, highlighting the importance of barrier assessment in disease evaluation.

### 5.3. Fluorescein Angiography

Fluorescein fundus angiography (FFA) is an imaging method [105]. It involves injecting sodium fluorescein into a vein and dynamically observing its distribution in the retinal blood vessels [106]. It is widely used to assess retinal vascular structure and permeability [95]. When the blood–retinal barrier is intact, fluorescein mostly stays within the blood vessels. In late-phase images, no obvious leakage should appear [95]. When the blood–retinal barrier becomes more permeable, fluorescein can leak out of the retinal vessels. This appears as diffuse or focal hyper fluorescence in the late-phase images [96]. As a result, the vascular leakage observed by FFA reflects functional abnormalities of the blood–retinal barrier, rather than purely structural changes [96]. In patients with uveitis, retinal vascular leakage often occurs during active inflammation. Its severity is closely related to the level of disease activity. Compared with static imaging, FFA can more sensitively reflect dynamic changes in barrier function. It has important value in assessing disease progression and treatment response [94]. Importantly, persistent fluorescein leakage may still be observed in some cases even when overt clinical signs of inflammation appear improved. This finding may indicate incomplete recovery of the blood–retinal barrier or ongoing subclinical vascular inflammation.

Accordingly, persistent FA leakage may reflect ongoing vascular instability despite apparent inflammatory improvement.

### 5.4. How Barrier Assessment May Influence Clinical Management

Beyond reflecting disease activity, assessment of blood–ocular barrier function may also provide clinically relevant information for disease management [2]. In particular, barrier-related parameters can help interpret situations in which conventional inflammatory indicators appear controlled, but underlying disease activity may persist [85]. For example, in some patients, inflammatory cell counts in the anterior chamber or vitreous may decrease following treatment, suggesting apparent clinical improvement. However, persistent abnormalities in barrier-related markers, such as increased aqueous flare or continued fluorescein leakage, may indicate incomplete restoration of barrier integrity and ongoing subclinical inflammation [85]. In such cases, barrier assessment may support the need for continued or intensified therapy rather than premature treatment tapering.

In addition, dynamic changes in barrier function may be useful for monitoring treatment response and guiding follow-up strategies. Improvement in barrier-related parameters may reflect stabilization of the intraocular microenvironment, whereas delayed or incomplete recovery may suggest a higher risk of disease persistence or recurrence. Therefore, integrating barrier assessment into longitudinal evaluation may provide complementary information beyond conventional inflammatory scoring.

Furthermore, barrier dysfunction may have potential value in relapse risk assessment. Although this role has not yet been fully validated, incomplete recovery of barrier function after apparent clinical remission may indicate residual pathological activity, which could predispose patients to future relapse. These findings suggest that barrier-related biomarkers may have potential value in relapse-aware disease monitoring.

**Figure 3:**
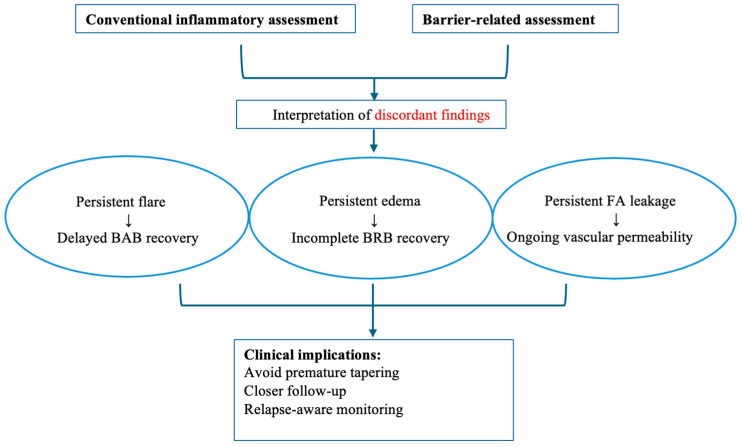
Barrier-based clinical interpretation framework in uveitis.

Taken together, assessing blood–ocular barrier function can provide additional information beyond conventional inflammatory indicators. The proposed barrier-based clinical interpretation framework is summarized in Figure 3. Barrier-related parameters may better reflect the actual disease status, especially when clinical signs appear improved but underlying abnormalities may still exist. Incorporating barrier assessment into routine evaluation may help improve disease monitoring and support more appropriate clinical decision-making.

Conventional inflammatory findings, such as anterior chamber or vitreous cells, may improve before full restoration of the intraocular microenvironment. Persistent barrier-related abnormalities, including aqueous flare, optical coherence tomography-detected macular edema, or fluorescein angiography leakage, may indicate delayed barrier recovery, residual subclinical inflammation, or ongoing vascular permeability abnormalities. Integrating these findings into clinical assessment may help avoid premature treatment de-escalation, guide follow-up intensity, and support relapse-aware management.

## 6. Challenges and Future Directions

Currently, indicators related to the blood–ocular barrier are mainly used to describe disease status, studies directly guiding treatment strategies remain limited. As a dynamic biological process, the functional status of the blood–ocular barrier may have different clinical implications at different stages of uveitis, providing an additional perspective for disease assessment and management. In the early stage of disease, when clinical symptoms may be mild or nonspecific, increased barrier permeability reflects alterations in the intraocular microenvironment and indicates a potential risk for disease onset or progression, thereby providing a basis for early intervention [6]. During active stage and treatment, changes in barrier function can serve as a supplementary indicator to evaluate treatment effectiveness. Improvements in barrier permeability may occur later than changes in clinical manifestations. Even when the number of inflammatory cells decreases or clinical symptoms improve, barrier permeability indicators may remain abnormal [5]. The clinical significance of barrier function measurement is often not fully recognized, possibly leading to an overly optimistic assessment of inflammation control [4]. Furthermore, during follow-up after clinical remission, incomplete recovery of barrier function may indicate that inflammation-related pathological processes are not fully resolved, and this may be associated with the risk of disease recurrence [107].

For example, in clinical practice, several commonly observed scenarios illustrate these points. In some patients, aqueous flare may remain elevated despite a reduction in inflammatory cell counts, suggesting delayed barrier recovery or residual subclinical inflammation. In other cases, fluorescein angiography may still show vascular leakage even when overt clinical signs appear controlled, indicating persistent vascular permeability abnormalities. Similarly, persistent macular edema may reflect incomplete restoration of the blood–retinal barrier despite apparent improvement in inflammatory activity. These examples highlight that barrier-related changes may provide important additional information beyond conventional inflammatory indicators.

From a therapeutic perspective, strategies targeting blood–ocular barrier dysfunction may be broadly divided into two categories.

First, anti-inflammatory therapies, such as corticosteroids, immunosuppressive agents, and biologics (e.g., anti-TNF or IL-6 blockade), may indirectly improve barrier function by reducing inflammatory damage [108,109,110,111]. While these approaches are effective in controlling acute inflammation, their effects on barrier restoration are often secondary and may not fully reverse established barrier dysfunction.

Second, emerging strategies may more directly target barrier integrity. These include modulation of signaling pathways involved in endothelial stability and tight junction regulation, such as JAK-related pathways, cAMP-mediated signaling, and the balance between Tie2 and VEGF pathways. These approaches aim to stabilize vascular permeability and restore barrier function more directly, although current evidence remains limited. The therapeutic implications of these anti-inflammatory and barrier-restorative approaches are summarized in Table 2.

Taken together, combining anti-inflammatory treatment with strategies that promote barrier restoration may provide a more comprehensive approach to disease management, and may help achieve more stable remission and reduce the risk of recurrence.

Clinically, it can also be observed that some patients continue to have vascular leakage or macular edema even after the number of inflammatory cells decreases [117], reflecting that the intraocular microenvironment has not fully returned to normal. Our review suggested that the vicious feedback between inflammation and blood–ocular barrier dysfunction may be an important reason for this problem. Therefore, “breaking the vicious cycle” and restoring blood–ocular barrier function could be a potential therapeutic goal of uveitis and other ocular inflammation. It may help achieve more stable and lasting treatment effects and reduce the risk of disease recurrence.

## 7. Conclusions and Perspectives

In summary, dysfunction of the blood–ocular barrier runs through the entire process of onset, progression, and recurrence of uveitis. Mechanistic studies show that inflammation can directly damage the structure and function of the blood–ocular barrier, while the dysfunction of barrier integrity further facilitates the entry of inflammatory mediators and immune cells into intraocular tissues. Together, these processes form a self-reinforcing cycle that contributes to disease persistence and progression. With advances in clinical detection techniques, the functional state of the blood–ocular barrier can now be dynamically assessed and is closely related to disease activity and prognosis. Barrier function is no longer only a subject of mechanistic research, but is increasingly recognized as a clinically relevant indicator for disease assessment and monitoring. Importantly, barrier assessment may complement conventional inflammatory evaluation by providing additional insight into disease activity, particularly in cases where clinical manifestations appear controlled but underlying microenvironmental instability persists. This may help explain apparently discordant clinical findings and supports a more relapse-aware approach to disease monitoring.

Based on the above insights, this review suggests that assessment and restoration of blood–ocular barrier function should be incorporated into the dynamic management and therapeutic goals of uveitis. This approach may provide a more comprehensive strategy for disease control. It may also complement conventional inflammatory assessment and help interpret apparently discordant clinical findings. Disrupting this self-reinforcing cycle may support more sustained disease control and a reduced risk of relapse.

## Figures and Tables

**Table 1:** Clinical methods for assessing blood–ocular barrier dysfunction in uveitis.

Method	Primary Readout	What It Reflects	Advantages	Limitations	Representative References
**Laser flare photometry**	Aqueous flare value	Breakdown of the blood–aqueous barrier	Non-invasive; quantitative; repeatable	Limited availability; affected by media opacity	[31,89,90,92]
**Optical coherence tomography (OCT)**	Retinal thickness; macular edema	Structural consequences of blood–retinal barrier disruption	Widely available; high spatial resolution	Indirect assessment: macular edema is nonspecific	[88,93]
**Fluorescein angiography (FA)**	Retinal vascular leakage	Dynamic permeability of retinal vessels	Sensitive to active inflammation	Invasive; qualitative or semi-quantitative	[94,95,96]

**Table 2:** Therapeutic implications of targeting blood–ocular barrier dysfunction in uveitis.

Therapeutic Approach	Main Target	Expected Effect on Barrier Function	Current Evidence	Clinical Implication	Representative References
**Corticosteroids**	Broad inflammatory pathways	Secondary improvement of barrier dysfunction through inflammation control	Established clinical use	Rapid control of active inflammation	[10,112]
**Conventional immunosuppressive agents**	Immune activation and cytokine production	Indirect stabilization of barrier function	Established use in non-infectious uveitis	Steroid-sparing maintenance therapy	[113]
**Anti-TNF therapy**	TNF-mediated inflammation and endothelial activation	May reduce cytokine-driven vascular permeability	Clinical and mechanistic support	Refractory or recurrent non-infectious uveitis	[114]
**IL-6 blockade**	IL-6-related inflammatory signaling	May improve inflammatory macular edema and barrier-associated leakage	Emerging clinical evidence	Refractory uveitic macular edema	[115]
**JAK-related pathway modulation**	Cytokine signaling and endothelial dysfunction	Potential direct protection of barrier integrity	Mainly experimental or emerging	Future barrier-restorative target	[116]
**cAMP-mediated barrier stabilization**	Endothelial tight junction regulation	Potential reduction in vascular permeability	Experimental evidence	Future adjunctive strategy	[51]
**Tie2/VEGF balance modulation**	Vascular stability and permeability	Potential stabilization of retinal vascular barrier	Disease-context dependent evidence	Future translational target	[54]

## Data Availability

No new data were created or analyzed in this study. Data sharing is not applicable to this article.
